# Active smelling in the American cockroach

**DOI:** 10.1242/jeb.245337

**Published:** 2023-11-08

**Authors:** Antoine Hoffmann, Einat Couzin-Fuchs

**Affiliations:** ^1^ Department of Biology, University of Konstanz, 78457 Konstanz, Germany; ^2^ Department of Collective Behavior, Max Planck Institute of Animal Behavior, 78464 Konstanz, Germany; ^3^ IMPRS for Quantitative Behaviour, Ecology and Evolution, 78315 Radolfzell, Germany; ^4^ Centre for the Advanced Study of Collective Behaviour, University of Konstanz, 78464 Konstanz, Germany

**Keywords:** Active sensing, Olfaction, Plume visualisation, Antennal movements, Cockroach

## Abstract

Motion plays an essential role in sensory acquisition. From changing the position in which information can be acquired to fine-scale probing and active sensing, animals actively control the way they interact with the environment. In olfaction, movement impacts the time and location of odour sampling as well as the flow of odour molecules around the olfactory organs. Employing a detailed spatiotemporal analysis, we investigated how insect antennae interact with the olfactory environment in a species with a well-studied olfactory system – the American cockroach. Cockroaches were tested in a wind-tunnel setup during the presentation of odours with different attractivity levels: colony extract, butanol and linalool. Our analysis revealed significant changes in antennal kinematics when odours were presented, including a shift towards the stream position, an increase in vertical movement and high-frequency local oscillations. Nevertheless, the antennal shifting occurred predominantly in a single antenna while the overall range covered by both antennae was maintained throughout. These findings hold true for both static and moving stimuli and were more pronounced for attractive odours. Furthermore, we found that upon odour encounter, there was an increase in the occurrence of high-frequency antennal sweeps and vertical strokes, which were shown to impact the olfactory environment's statistics directly. Our study lays out a tractable system for exploring the tight coupling between sensing and movement, in which antennal sweeps, in parallel to mammalian sniffing, are actively involved in facilitating odour capture and transport, generating odour intermittency in environments with low air movement where cockroaches dwell.

## Teaser

**Summary:** Antennal movements in the American cockroach highlight the active role of antennal motion in olfaction via efficient asymmetric sampling strategies, high-frequency oscillations and alteration of the odour environment structure.

## INTRODUCTION

Olfaction is crucial in almost every aspect of life: finding food and assessing its quality, avoiding danger, communication and mating (Ache and Young, 2005; Hansson and Stensmyr, 2011). Odour molecules are transported from their sources by currents of air (or water) that vary in flow and turbulence. Both the physical properties of the airflow (or other carrying media) and the chemical attributes of the odour, such as volatility (Cometto-Muñiz et al., 2003), impact its dispersal, often creating complex spatio-temporal concentration distributions (Moore and Crimaldi, 2004; Murlis et al., 2000; Pannunzi and Nowotny, 2019). Nevertheless, despite the structural complexity of natural odour landscapes, animals successfully use information from these concentration distributions to track odour plumes (Murlis et al., 1992; Vickers, 2006), localise their sources and segregate concurrent odours that arise from different objects (Baker et al., 1998; Sehdev and Szyszka, 2019). An important aspect of olfactory behaviour, as commonly acknowledged in the recent literature, is the active nature of interactions between the sensing body and the olfactory environment (Crimaldi et al., 2021; Wachowiak, 2011). Examples of such interactions are exhibited in a variety of distinctive odour-guided behaviours such as casting across plume edges (Kennedy, 1983; Willis and Baker, 1984), sniffing (Wachowiak, 2011), antennule movements (antennule ‘flicking’ in crustaceans: Koehl et al., 2001; antennal movements in insects: Claverie et al., 2023; Huston et al., 2015; Lei et al., 2022) and wing fanning (Li et al., 2018). A full mechanistic understanding of these interactions and their role in olfactory processing is still in the early stages, however, especially when we compare it with the highly detailed knowledge on the olfactory circuits themselves (Galizia and Rössler, 2010). To this end, we note the importance of tractable model systems in which the bidirectional interactions between motion and sensing could be investigated in detail. Utilising an insect preparation with a well-described olfactory system and highly mobile antennae, the American cockroach, *Periplaneta americana*, we studied how the olfactory environment impacts, and is impacted by, antennal movements.

Cockroaches are mainly ground-dwelling insects that favour humid, confined spaces in low light conditions. Their exceptionally long and mobile antennae, covered with olfactory sensilla distributed along the entire length, provide a wide working range for odour detection (Lockey and Willis, 2015). Odour information is transmitted into the antennal lobe where information about odour identity and quantity can be evaluated based on the population of activated glomeruli (Wilson, 2013; Watanabe et al., 2010; Fuscà and Kloppenburg, 2021). Less is known about how the spatial structure of the odour environment is encoded. Presumably, both bilateral comparisons of antennal inputs (‘tropotaxis’: Borst and Heisenberg, 1982; Duistermars et al., 2009) and temporal evaluation (‘klinotaxis’: Lockey and Willis, 2015; Willis and Avondet, 2005) are involved. An antennotopic neural arrangement that allows spatial mapping along the insect antenna, even without movement, has so far only been shown for the specialised pheromone-processing macroglomerulus pathway (Nishino et al., 2018; Paoli et al., 2020). It is unknown, however, whether cockroaches are able to behaviourally utilise this neural organisation for navigation and/or whether similar antennotopic organisations exist for other odorants as well (Nishino et al., 2018; Paoli et al., 2020).

Spatial evaluation and successful source localisation of all odours, nevertheless, rely on successive behavioural sampling. These typically follow a reactive method – in which odour encounters elicit a switch in behaviour (e.g. from crosswind casting to up-wing surging; Cardé and Willis, 2008) – and/or a strategic one – where odour information is integrated over time (Pang et al., 2018; Voges et al., 2014). When navigating toward distant odour sources, cockroaches utilise wind direction information and exhibit casting movements across the lateral boundaries of wind-borne plumes (Willis and Avondet, 2005; Willis et al., 2008) in a similar manner to odour-source tracking in flying insects (Talley et al., 2022). Motivated to further understand how, at the local scale, the antennae actively participate in sampling and mapping the olfactory space, we studied antennal kinematics during static and moving odour encounters.

This paper is structured as follows: we start with a characterisation of spatial antennal sampling to investigate whether and how antennal movements are modified with respect to the location and nature of surrounding odorants. Our working hypothesis is that, as in other types of search behaviours (Mehlhorn et al., 2015), exploration– exploitation trade-offs also hold for antennal movement when scanning an odour space. We therefore expect efficient scanning strategies to include both episodes of localised movement, focused on a position of interest, and also wide-range scanning of the rest of the available space. We then continue to characterise the fine-scale temporal dynamics of antennal oscillations and discuss the relevance of the observed odour-driven changes for odour transduction and intermittent sampling. The final part of our paper focuses on the impact antennal movements have on the olfactory environment itself.

## MATERIALS AND METHODS

### Experimental setup

**Fig. 1.**
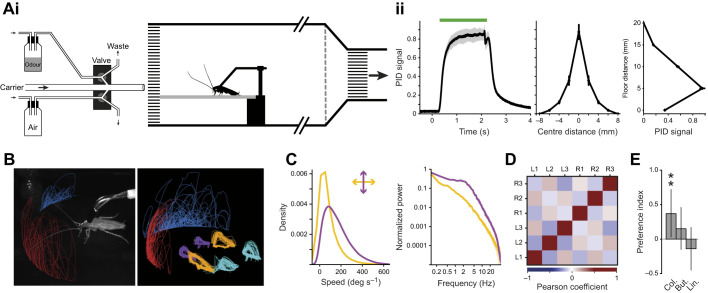
**Wind tunnel setup to test active cockroach olfaction.** (Ai) Schematic diagram of the odour delivery device (Raiser et al., 2017) and wind tunnel. (Aii) Odour stimulus. Left: normalised photo-ionisation detector (PID) signal as a proxy for the amount of odour molecules for a 2 s pulse (green bar). The delay between the valve trigger and the onset of the odour was compensated for in every subsequent analysis. Middle: PID signal for the width of the odour pulse. Right: PID signal for the height of the odour pulse. Means±s.e.m.; *N*=5 trials. (B) Left: view from one of the cameras from the 3D tracking system of a male cockroach in the wind tunnel. The animal was free to move its head and limbs on a slippery glass surface. Right: 3D tracking example for the antennae tips, head, and leg movements. (C) Overall analysis of antennal kinematics from pooled trials shows movement to be generally slower in azimuth (yellow) and faster in elevation (purple). Plotted are the distributions of angular antennal speeds with the corresponding Fourier spectra (*N*=100). (D) Pairwise correlation matrix computed from the frame-to-frame speed of each leg and pooled from all trials. R1–R3 and L1–L3 represent front to hind right and left legs, respectively (Pearson's correlation coefficients; *N*=330). (E) Preference index (PI) computed from arena choice assays between an odourised shelter and a control shelter: PI=(Time_odour_−Time_control_)/Time_total_ (*N*=15 for colony odour, *N*=19 for butanol and linalool). Asterisks (**) indicate differences from zero with ≥95% certainty.

Adult males of the American cockroach, *Periplaneta americana* (Linnaeus 1758), were kept on a 12 h:12 h light:dark cycle at 24°C and 65% humidity in the animal facility of the University of Konstanz (Germany). Male cockroaches were collected and were briefly immobilised through cold treatment to facilitate handling. Animals were tethered on a glass surface in a 20×20×100 cm Plexiglas wind tunnel (custom design; Fig. 1A), using a thin ‘leash’ of a plastic tube wrapped around the thorax and attached to a metal rod for fixation. Care was taken to place the animal in a natural position that allows flexible stepping on a glass surface rendered slippery with a thin layer of glycerol. Behavioural experiments started after 20 min of acclimatisation. We performed three experimental protocols for a given animal: a static frontal odour stimulus centred on the animal's head (‘position 1’); a static frontal stimulus on the side, 3 cm from the midline, which is well within antennal reach (‘position 2’); and a moving frontal stimulus shifting from position 1, via position 2, to the very edge of the antennal reach (‘position 3’). Three odours were delivered in a pseudo-randomised order (colony odour, butanol, linalool).

The wind tunnel was designed to create a laminar air flow for delivering spatially and temporally precise odour stimuli. It includes a rotary fan placed in the exhaust tubing to create an air suction which is evacuated to the laboratory ventilation system. To reduce turbulence caused by the fan, a 2 cm thick aluminium honeycomb mesh and a net were placed at the end of the tunnel. An additional honeycomb at the entrance of the tunnel ensured a laminar air flow in the experimental recording area (see Fig. 1Ai). The air speed was set at 0.15–0.2 m s^−1^ for all behavioural experiments, far below the typical running speed of the American cockroach.

Odour valence assays were done as in Günzel et al. (2021). In brief, we used a custom circular PVC arena (90 cm diameter) closed with a Plexiglas plate. Odours were passively delivered via small holes in the floor. A uniform overhead LED light illuminated the arena and red transparent shelters with open sides were placed above the odour location and its opposite control location. A single camera (acA2040–90 μm; Basler AG, Ahrensburg, Germany) recorded the arena from above. Recordings started 5 min after the odour and animal were placed in the arena, and lasted for 30 min. A preference index (PI) between the odourised shelter and a control shelter was computed:
(1)

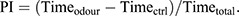


### Odours

Odours were delivered via a custom-made olfactometer based on Raiser et al. (2017) which allows stable, flow- compensated delivery of odorants for extended periods of time. Three odours were presented to the animals: butanol (Sigma-Aldrich, product code: 19422), a compound found in rotting food as a product of fermentation processes; linalool (Sigma-Aldrich, product code: L2602), a floral compound; and a ‘colony extract’ collected from the substrate, faeces and debris in the rearing colonies, which is a mixture of faeces odorants, pheromones and other odorants from conspecifics. For the arena valence assays, the colony extract had to be concentrated into small vials to accommodate the setup and we therefore used an extraction made by Hiroshi Nishino, Hokkaido University, Japan. Odour dilutions (10^–2^ for butanol and 10^–1^ for linalool) in mineral oil (Acros Organics, product code: 124020010) were chosen because of their overall similar strength of response in the cockroach antennal lobe (Paoli et al., 2020, and unpublished data); 200 µl of butanol and linalool were placed in 20 ml vials (Schmidlin Labor+Service GmbH, product code: 5520090873) sealed with Teflon septa (Schmidlin Labor+Service GmbH, product code: 5520030142) to be used for delivery and all odorants were regularly renewed to avoid depletion. The stimulus sequences were pre-programmed for accurate repeatability in custom LabVIEW software (National Instruments, Austin, TX, USA) which controls a compact RIO system equipped with a digital I/O module NI-9403 (National Instruments). For experiments with a moving stimulus, the outlet of the delivery device was shifted laterally on a rail via an Arduino-actuated stepper motor and a custom-written script, synchronised with the odour stimulus sequence. The profile of odour distribution in the wind tunnel was tested with a photo-ionisation detector at its lowest pump setting (Mini-PID model 200A, Aurora Scientific Inc., Aurora, ON, Canada; recordings with Spike2 software, Cambridge Electronic Design Limited, Cambridge, UK) to ensure a sharp onset and offset in time and space (sharp rise and decay times with a stable plateau within, and a narrow stimulus width; Fig. 1Aii).

### Recording and tracking

The experimental apparatus was placed in full darkness, illuminated only with infrared (IR) lights (850 nm wavelength, model IN-907, Instar, Frankfurt, Germany) to avoid visual cues. Recordings were made via the Motif system developed by LoopBio (Loopbio GmbH, Vienna, Austria; http://loopbio.com/recording/). Three calibrated and synchronised Basler cameras (Model acA1920-150um, Basler AG, Ahrensburg, Germany; lenses by Kowa Optimed GmbH, Düsseldorf, Germany) equipped with IR long-pass filters were placed to capture the animal from three different views at 100 frames s^−1^. Uniform lighting conditions for the video recordings were obtained by arranging IR lights (850 nm wavelength) around the recording area. Tracking and 3D reconstruction of the animal's legs, head and antennae tips were achieved with the LoopBio software ‘Loopy’ (http://loopbio.com/loopy/). The tracking data were then further processed with custom-written scripts in R (version 4.2.0., https://cran.r-project.org/). All metrics and analyses were calculated from these 3D coordinates in R.

### Behavioural data analysis

Antennal angles were computed by approximating each antenna as a straight line from the tracked head point to the tracked antennal tip (cockroaches cannot voluntarily bend their antennae and the passive bending in the air is negligible). In the horizontal plane (azimuth), the angle between these lines and the animal's head-line (a virtual line through the animal's tracked head point) was calculated, such that 0 deg corresponds to an antenna exactly parallel to the head-line. Negative angles correspond to the left and positive angles to the right. In the vertical plane (elevation), the angle between that same virtual horizontal head-line and the approximated antenna was calculated such that negative values correspond to the tip being below, and positive values to above, the head-line. The heading direction corresponds to the angle of the head-to-tether line to the midline passing through the fixed tether point at the thorax (negative values correspond to the left, positive value to the right, as for the antennal angles). The absolute heading corresponds to the absolute value of that angle, quantifying the deviation from the midline, instead of heading direction.

The walking behaviour was derived from the tracked tarsi coordinates. For computing the stepping rate, we averaged the step rate of the front and middle legs (L1–2 and R1–2). We only took these four legs into account as they were reliably tracked and showed representative read-outs of walking activity (L1–2 and R1–2 were visible on most frames of each of the recording views, whereas the hind legs tended to be obstructed by the animal's body or tether more often). Single steps of each leg were identified as peaks in the *y*-coordinates (forward motion) and the instantaneous stepping rate was computed and normalised. The final stepping rate over time for a given trial thus corresponds to the mean normalised instantaneous stepping rate of the four front legs at any given recorded frame.

Antennal coordinates were normalised to compare antennal positions across trials and animals. Although head movement is relatively small, we centred the *x* and *y* coordinates (left–right and back–front axes, respectively) around the head point at each time point such that (*x*,*y*)=(0,0) corresponds to the head position on the horizontal plane. The *z* coordinates (vertical axis) were centred as well, such that zero corresponds to head level. Normalised coordinates were calculated by scaling to the respective maximum absolute value within a trial, effectively normalising by the antennal length of each animal. The normalised metrics were calculated within this new coordinate system, and thus expressed as a proportion of the antennal length (antennal lengths of our test animals approximately ranged from to 45 to 55 mm).

Spatial heat maps of antennal tips were generated from the centred coordinates, normalised by the maximum possible coordinate across trials in the dataset. The (*x*,*y*,*z*)=(0,0,0) position in each heat map thus corresponds to the head position and coordinates of 1 and −1 are the furthest reached point across trials. The sum of observations of antennae tip positions across trials was computed in 24 bins for each axis. The heat maps were then individually normalised by their overall observation sums for comparison. Density curves correspond to the empirical kernel probability density function (density function from the base stats package in R; the same bandwidth parameter value was used for the curves of a given panel). The area under each curve thus equals 1.

The distance to the stream was first calculated for each time point using the absolute Euclidean distance between the normalised antennal tip coordinates and the centre line of the stimulus stream. The antennal range corresponds to the absolute maximum difference between horizontal positions (*x* coordinates) of each individual antenna. The overall range corresponds to the absolute maximum difference between the left-most and the right-most positions of the two antennae combined.

For the aforementioned metrics, odour-induced effects were calculated by comparing metrics between the odour time window and a time window of the same length directly before the odour onset. This was done by calculating the difference of the metric at a given time point to the mean of the pre-stimulus window (i.e. ‘changes’ in a given normalised metric), within trial. For the shifting stream experiment, we had a sham control with a non-odorised air stream for each animal. In addition to the pre-stimulus baseline, we corrected the metrics of each odour-stimulus trial with the values of the sham control trial of the same animal. For the statistical models of overall odour-induced changes, the time-varying differences from the baseline were averaged in the windows of interest (pre-stimulus averages are thus at zero and were not included in the models).

The average frequency spectra analysis in Fig. 1 are based on a fast Fourier transform (*spec* function, *seewave* package in R; Sueur et al., 2008). The more detailed spectral analysis used to investigate high-frequency antennal movements is based on a continuous wavelet analysis (*analyze.wavelet* function, *WaveletComp* package in R; https://CRAN.R-project.org/package=WaveletComp). The continuous wavelet power spectra of angular time series were averaged in 1 s time bins and pre-defined frequency bands {[0.5–1), [1–2), [2–3), [3–5) and [5–10) Hz}. The power values for each frequency band were then scaled as follows:
(2)

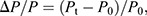
where *P*_t_ is the power of a given time bin and *P*_0_ is the average power of a given frequency band in the pre-stimulus window. The resulting metric thus quantifies changes in wavelet power for each frequency band as a proportion of the pre-stimulus average.

### Visualising air-borne plumes

TiO_2_ smoke resulting from the contact of TiCl_4_ (Fluka, CAS 7550-45-0) with air humidity was used to visualize the air flow from the odour delivery device at a low wind speed (0.05 m s^−1^) (Fig. S4A). A planar IR laser (0.2 mm light sheet thickness, 808 nm wavelength, 7 W; Laserwave, Beijing, China) was used to highlight a horizontal section of the smoke and reveal its flow patterns. Cockroaches were then placed in the wind tunnel, in an analogous manner to the odour experiments. A top view of the laser-illuminated region was recorded with the top single Basler camera of the Motif system at 100 frames s^−1^. Sequences of interest, where an antenna moved through the smoke, were isolated for analysis. For the condition with a static antenna, a recently dead animal was used.

For the analysis of smoke distribution, selected frames were processed in R and Adobe Photoshop. We carefully hand-drew a mask on the animal using the original greyscale image in order to have a silhouette. We thresholded the original greyscale images with the same threshold value for all frames of a given sequence and defined a region of interest (ROI) for analysis in front of the head. We then applied the mask to the thresholded images in order to remove pixels representing body parts and antennae. This process was done manually, resulting in cropped images where white pixels only represent smoke particles. We then calculated the number of white pixels for each row of the ROI (in R, https://cran.r-project.org/), giving us the left-to-right distribution of smoke particles in front of the animal.

### Odour fluctuations and antennal movements

As a proxy for the relative odorant concentration over time during antennal movements, the photo-ionisation detector (PID) sensor was placed at head level, just behind the left antenna at about 2/3 its length in its natural resting position. The PID pump was set to its lowest setting and air speed was set to 0.05 m s^−1^. The odour stream was centred between the head and the sensor (at this location and air speed, baseline fluctuations were more pronounced than in Fig. 1). The odorant used was pure butanol (200 µl in a 20 ml vial), as it is easily detected by the PID (butanol ionisation potential=10.04 eV). Tracking of the head and antenna tip was achieved with the loopy software (LoopBio GmbH, http://loopbio.com/loopy/), from a top view. Azimuth and elevation angles were used to compute the combined angular antennal speed in R.

To analyse the correlation between antennal movements and odour fluctuations, we systematically selected 2 s windows, according to local peaks in antennal speed, resulting in a wide range of antennal movement regimes (local peak detection was based on the smoothed speed via a moving average, but the unaltered speed was used for the analysis). As a control, windows of the same size were randomly selected from recordings with the antenna fixed at its base. Odour fluctuations were estimated from the rate and magnitude of the PID voltage peaks. All calculations for peak detection, fluctuation rate (average instantaneous frequency of all detected PID minima and maxima) and magnitude (average voltage difference between minima and their subsequent maxima – positive contrasts) were performed in R.

### Statistical analysis

Throughout, we used Bayesian statistics based on Markov Chain Monte Carlo sampling [using the packages *brms* (Bürkner, 2017) and *cmdstanr* (https://mc-stan.org/cmdstanr/, https://discourse.mc-stan.org), implementing *Stan* in R] in combination with methods from Korner-Nievergelt et al. (2015). We used weakly informative priors from the *brm* model fitting function. All model outputs are presented in the scale of the original data and were thus back-transformed if any transformation (pre-transformation and/or link transformation) was used to fit the model. Estimates correspond to the 50% quantile of the posterior distributions (mean), with the 2.5% and 97.5% quantiles as 95% credible intervals. Certainty of odour-induced effects and comparisons were tested by calculating the proportion of posterior samples that were above or below a reference value of either zero (i.e. the pre-stimulus baseline), a control value or another within-model group. Differences with ≥90%, ≥95% and ≥99% certainty are marked with asterisks (see figure legends for details on the comparisons made in each). Table S1A summarises the model parameters for each analysis.

## RESULTS

### Movement kinematics are altered during odour exposure

Antennal kinematics were measured under laminar flow conditions in our wind tunnel (Fig. 1Ai) in order to characterise whether and how movement dynamics are changed according to the olfactory environment. Overall, we performed 215 trials on 31 animals, tracking their legs, head and antennal tip positions in 3D (Fig. 1B) to characterise their kinematics. The animals were tested after acclimation and in a calm state, showing spontaneous slow and intermittent walking bouts (average stepping rate of about 2 Hz when walking). The general analysis, pooling trials and conditions, revealed continuous horizontal antennal sweeping that included relatively large and slow cycles (Fig. 1C) and a typical walking gait for slow walking (approximating a double tripod gait; Fig. 1D). We found no correlation between stepping rate and antennal speed in our data (not shown). Animals were tested with three test odours delivered in different positions. The set of odours, comprising an innately attractive colony extract mixture, a mildly appetitive short-chain alcohol (butanol) and a neutral to mildly aversive floral odorant (linalool), were chosen as a relatively broad set of odours to highlight both common features and differences in observed responses (valences based on a preference test to the three odours is shown in Fig. 1E).

**Fig. 2.**
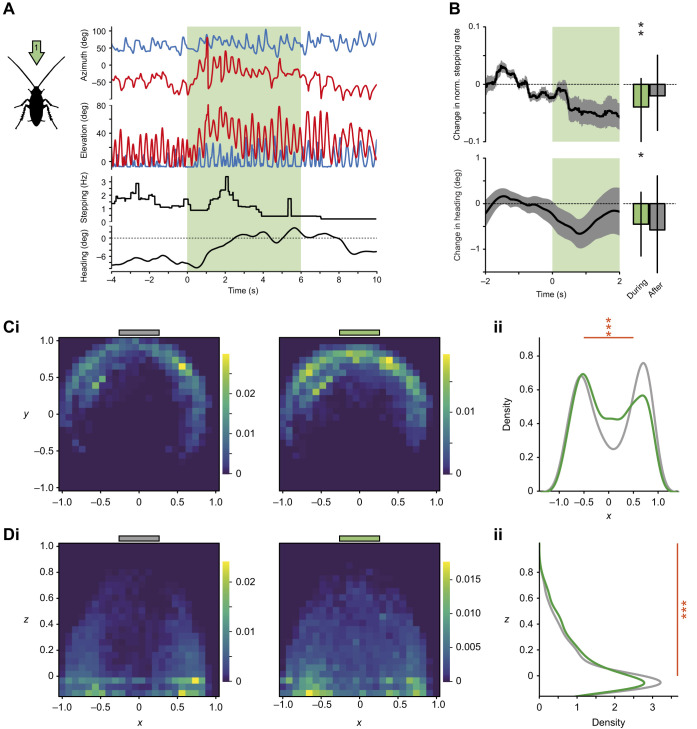
**General odour-induced behavioural responses.** (A) Left: schematic view of the stimulus in position 1 (centre). Right: single trial example with time series for antennal angles in azimuth and elevation (left and right antennae in red and blue, respectively), mean stepping rate and heading angle (smoothed; negative and positive values indicate left turn and right turn, respectively). The shaded green time window indicates the odour stimulus. (B) Top: change in normalised stepping rate with colony odour, compared with the pre-stimulus air (*n*=40 trials, *N*=29 animals). Bottom: change in absolute heading, compared with the pre-stimulus heading (negative values indicate that the animals are moving their heads towards the stimulus; *n*=47 trials, *N*=30 animals; mean±s.e.m. time series and model means with credible intervals). Asterisks indicate the certainty levels for the difference to zero (*≥90%, **≥95%). (C) Horizontal antennal tip distributions before and during a colony odour stimulus (2 s windows). (i) Presence density maps in the normalised coordinate system (zero=head position, ±1=maximum coordinate across trials). Colours indicate relative proportions of observations per pixel. Grey (before; left) and green (during; right) bars above indicate the location of the air/odour stimulus. (ii) Antennal tip distributions on the *x*-axis (left-to-right) before and during the odour stimulus (grey and green curves, respectively). (D) Vertical antennal tip distribution before and during a colony odour stimulus for the same trials. (i) Presence density maps in the normalised coordinate system (zero=head position, on average 5 mm above ground, +1=maximum coordinate across trials). Colours indicate relative proportions of observations per pixel. Grey (before; left) and green (during; right) bars above indicate the location of the air/odour stimulus. (ii) Antennal tip distributions on the *z*-axis (ground to top) before and during the odour stimulus. In C and D: *n*=44 trials, *N*=24 animals. Asterisks indicate the certainty levels for the difference in odour means from the air control in the zones defined by the orange bars (*≥90%, **≥95%, ***≥99%).

Shortly after odour presentations, changes in legs and antennae kinematics were observed. A single trial example of antennal movements (horizontal and vertical components of the left antennal tip) and the corresponding stepping rate and heading direction during a 6 s odour stimulus (colony, central position 1) are shown in Fig. 2A (see also Movie 1 for this trial). As seen here and in most tested animals, shortly after odour onset, animals slowed down leg movements and centrally aligned their heading direction. Fig. 2B shows time traces and binned averages for trials with the colony odour. Responses for the other tested odours (see Fig. S1B,C) showed a similar decrease in leg movements but no consistent immediate change in heading. In parallel, general changes in antennal position were observed in both azimuth and elevation (Fig. 2C,D; post-stimulus period shows intermediate positioning between the pre-odour window and the odour response in Fig. S2D). In the absence of odour, the antennae sweep around their natural position of approximately 50 deg from the midline, covering a wide range with a relatively small overlap at the centre (left heatmap in Fig. 2Ci and grey density curve in Fig. 2Cii). When an odour is presented around the midline position (example in Fig. 2A, stimulus in position 1), this overlap increases (Fig. 2Ci right heatmap and green density curve in Fig. 2Cii). In addition, the antennal sweeps, which are mostly confined to ground level in the absence of odour, show drastic increases in elevation, covering a larger vertical space with more frequent upstroke movements (Fig. 2D). Note that our odour stimulus did not surpass 2 cm in height and was strongest at 5 mm (about head height, Fig. 1Aii), which is still far below the height that the antennae can reach (about 5 cm from the ground). This behaviour was not odour specific, with similar changes in coverage observed for the other tested odours (see Fig. S2 and the following analyses).

### Trade-off between local sampling and large-scale scanning

**Fig. 3.**
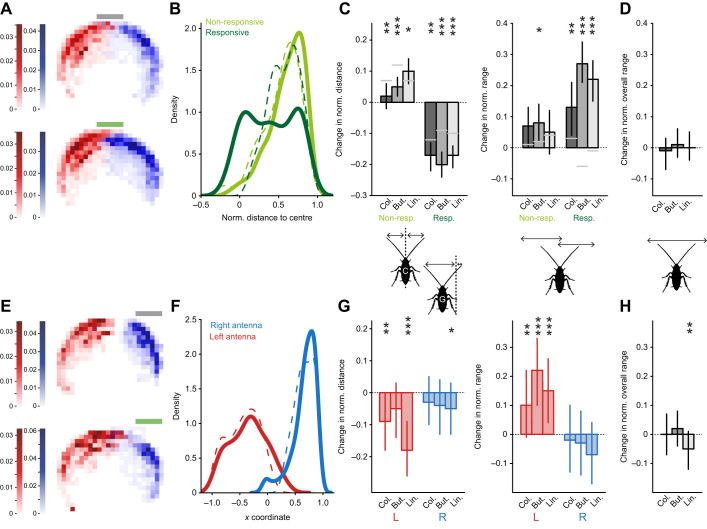
**Trade-off between local sampling and global scanning, and asymmetries in antennal movement.** (A) Horizontal antennal tip distributions before (grey; top) and during (green; bottom) a colony stimulus, delivered centrally in position 1 (red and blue indicate left and right antennae, respectively). Same data as in Fig. 2C. (B) Distributions of the horizontal distances between the antennal tip and the stimulus centre. In each trial, the antennae were labelled as ‘responsive’ and ‘non-responsive’, based on their movement towards the odour. Dashed lines correspond to the air control, solid lines correspond to the distributions during the odour stimulus (here, colony odour). (C) Left: odour-induced changes in distance to position 1 for each antenna (responsive and non-responsive) and each odour (means and credible intervals). Right: odour-induced changes in antennal range for each antenna (responsive and non-responsive identities transferred from the left panel) and each odour (model means and credible intervals). Grey lines indicate baseline control changes. Asterisks indicate the certainty levels for the difference in means from their respective control (*≥90%, **≥95%, ***≥99%). (D) Changes in overall antennal range for the three tested odours (means and credible intervals; *n*=44 trials, *N*=24 animals for colony odour; *n*=46, *N*=25 for butanol; *n*=48, *N*=25 for linalool). (E) Same as in A, but for an odour stimulus in position 2 (side stimulus). (F) Distributions of left (contralateral, red) and right (ipsilateral, blue) antennal tips during pre-stimulus air (dashed lines) and odour stimulus (colony odour, solid lines). (G) Left: odour-induced changes in distance to position 2 for each antenna and each odour (model means and credible intervals). Right: odour-induced changes in antennal range for each antenna and each odour (model means and credible intervals). (H) Odour-induced changes in overall antennal range for each odour (model means and credible intervals). In G and H, asterisks indicate the certainty levels for the difference in means from zero (*≥90%, **≥95%, ***≥99%) with *n*=20 trials, *N*=11 animals for colony odour and butanol; *n*=21, *N*=11 for linalool. Considered window sizes were 2 s for the pooled trials in A–D and 4 s for E–H.

Analysing the movement data in more detail revealed that even though the two antennae had equal access to the odour in a symmetric stimulus configuration (static position 1, Fig. 3A–D), we observed asymmetrical antennal responses. In order to investigate inter-antennal response differences, we compared odour-induced responses of each respective antenna to a baseline movement asymmetry in the absence of stimulus. We defined the ‘responsive’ antenna for each trial as the one with the strongest decrease in distance to the stream upon odour onset (regardless of its left/right identity). As a control, we applied the same method to a baseline variation between two consecutive time windows before odour onset (only clean air) and without transferring antenna identities (grey lines in Fig. 3C). This showed that the increase in activity predominantly occurred in one of the two antennae [spontaneously responsive antenna; Fig. 3B: low level of pre-stimulus asymmetry (dashed curves) is increased during the odour presentation (solid curves); and Fig. 3C: odour-induced decrease in distance to position 1, with no consistent side bias across trials (Fig. S1B)]. Note that, as the stream was centred, the proximal ends of both antennae were in contact with the odour throughout the trials and no relationship between the change in antenna tip distance to position 1 and its raw distance at odour onset was found.

In addition, the identity of the antenna labelled as responsive in terms of distance to the stream was transferred for the antennal range calculation, which was compared with its antenna-specific baseline variation instead of a neutral baseline. In the majority of cases, the same responsive antenna also increased its sweeping range (Fig. 3C: odour-induced increase in range), which resulted in a longer time spent around the odour without compromising the overall sensory space. Fig. 3D shows that the overall space covered by both antennae was unaffected.

To test whether this behaviour is specific to the central location, a similar analysis was carried out with a second set of trials in which the odour stream was positioned on the right side of the animal (Fig. 3E–H, position 2). In this position, the contralateral antenna (in red) cannot reach the stream. The left/right identities were maintained for quantifications and thus compared with their own pre-odour baseline movement. As for position 1, when an odour was presented, the antennae spent more time overall close to the odour (Fig. 3E–G). Here, the contralateral antenna increased its sweeping range, thus reaching further towards the odour side, while the ipsilateral one (in blue) tended to decrease its range around the odour (Fig. 3G). Altogether, however, as for the central odour, the overall scanning range covered by both antennae was mostly maintained, for all three tested odorants (with a slight decrease in range for position 2 with linalool, Fig. 3H). This represents an effective space coverage, in which, via asymmetric scanning behaviour (spontaneous in position 1 and experimentally imposed in position 2), an increased odour exposure is achieved without a loss in exploration range for both stream positions.

### The antennae follow a moving odour stream

**Fig. 4.**
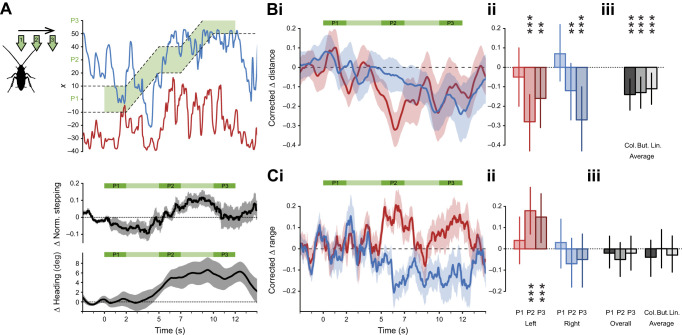
**Stream-following behaviour.** (A) Left: schematic view of the stimulus shift with three static periods in position 1 (P1, centre), position 2 (P2, side) and position 3 (P3, extreme side). Top right: example trial of antennal tip positions on the *x*-axis (left to right) over time. Dashed lines delineate the location of the stream. Green shading represents the time of the odour stimulus (here, colony odour). Middle: change in normalised stepping rate over time (mean±s.e.m.). Bottom: change in heading direction (left to right) over time (mean±s.e.m.). (B) Changes in distance of antennal tip positions to the stimulus centre. Negative values indicate larger displacements towards the odour compared with the air control. (i) Time series of control-corrected changes in distance to the moving stream centre for both antennae for the colony odour (means±s.e.m., smoothed with a moving average window of 1 s). (ii) Model means and credible intervals of control-corrected changes in distance for each antenna in the static positions P1–3 (colony, same scale). (iii) Overall model means across P1–P3 with credible intervals for each odour (same scale). (C) Changes in antennal range. Positive values indicate larger ranges than for the air control. (i) Time series of control-corrected changes in range for both antennae for colony (means±s.e.m., smoothed with a moving average window of 1 s). (ii) Model means and credible intervals of control-corrected changes in range for each antenna in the static positions P1–3 (colony odour, same scale). (iii) Left: model means of overall range (total range of the two antennae combined) and credible intervals for the static positions for colony. Right: overall model means across P1–3 and credible intervals for each odour (same scale). *N*=13 for each odour. In B and C, asterisks indicate the certainty levels for the difference in means from zero (**≥95%, ***≥99%).

To further test these findings, we monitored antennal movement during moving stimuli (Fig. 4), starting from the centre position 1, via the intermediate position 2 (side stimulus in Fig. 3) to the very edge of the animal's reach (dashed white/green area in Fig. 4A shows the air/odour positions for this experimental set, noted also as P1–P3). To differentiate between mechanical and odour-induced effects, all responses of odour trials were compared with sham air controls. Analysing body movement revealed significant odour-following behaviour in heading direction (bottom panel in Fig. 4A shows that the average heading direction follows the stream movement in colony trials), and in walking behaviour (middle panel in Fig. 4A shows that the initial decrease in speed with odour presentation in P1 changes to increased stepping rate when the odour is shifted). This following behaviour was odour specific, with clear following of colony odour and butanol, but no following with the air control and linalool (Fig. S3). This was reflected by the antennal activity, in which clear following behaviour in both antennae was observed for the attractive odours (significantly higher correlation between the movement of the antennae and the stream in the presence of an odour compared with the air control, see example in Fig. 4A and Fig. S3C for correlation coefficient values). As shown by the example in Fig. 4A and the time course averages (Fig. 4Bi) and binned averages (Fig. 4Bii), with odour presentation, both antennae shifted their positions along with the stream's movement such that a small distance to the odour was maintained throughout (Fig. 4Biii). Note that the antennal shifts were most prominent at the positions in which they fully lost contact with the odour (P2 for the contralateral antenna and P3 for the ipsilateral one), potentially as a means to regain contact at the tip. As was the case for the static stream in P2 (see Fig. 3), the sweeping range of the contralateral antenna (red) increased while the range of the ipsilateral one tended to decrease here as well (Fig. 4Ci,ii), with the overall range covered by both remaining unaffected (Fig. 4Ciii).

### High-frequency antennal sweeps increase during odour encounter

**Fig. 5.**
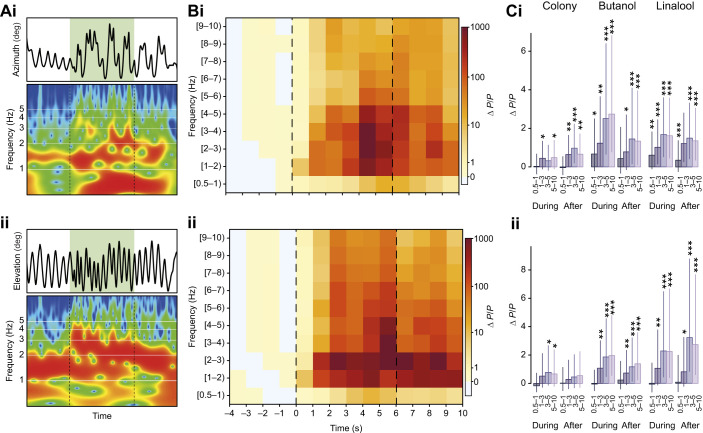
**Odour-induced high-frequency antennal movements.** (A) A single trial example with time series and its corresponding wavelet transform for the antennal angles in azimuth (i) and elevation (ii). Shown is a truncated log frequency range of 0.5–10 Hz. Green shading indicates the time of a colony odour stimulus, delivered in position 1 (centre). (B) Mean change in wavelet power, scaled for each frequency band by its pre-stimulus average of antennal angles in azimuth (i) and elevation (ii). Vertical dashed lines indicate odour onset and offset (colony odour). (C) Corresponding model means and credible intervals of scaled power changes (Δ*P*/*P*), during and after the odour stimulus for each odorant. For the analysis, frequency bands were pooled into four frequency ranges, and the analysis for the horizontal (i) and the vertical (ii) movement components is shown separately. Colony odour: *N*=24 for azimuth, *N*=14 for elevation; butanol: *N*=13 for azimuth and elevation; linalool: *N*=26 for azimuth, *N*=14 for elevation. Asterisks indicate the certainty levels for the difference in means from zero (*≥90%, **≥95%, ***≥99%).

To further characterise antennal spatio-temporal dynamics during odour encounters, we applied a wavelet analysis, decomposing the time series of azimuth and elevation angular antennal positions into time–frequency space (an example of horizontal and vertical movement traces with their respective wavelet decompositions is given in Fig. 5Ai,ii; see Materials and Methods for more details). This allowed quantification of changes in sweeping patterns at multiple temporal scales simultaneously. As illustrated in the example trace (Fig. 5A) and population average (Fig. 5B,C), odour presentation was associated with an increase in power in all frequency bands above 1 Hz, in both azimuth and elevation for all tested odours. The increase in high-frequency movement bouts lasted on average for the entire duration of the odour stimuli. Notably, the increase in horizontal and vertical sweeping persisted after odour offset (horizontal sweeps increased even further for the colony odour) and this was consistent across odours (Fig. 5Ci,ii).

### High-frequency antennal movement impacts odour distribution

**Fig. 6.**
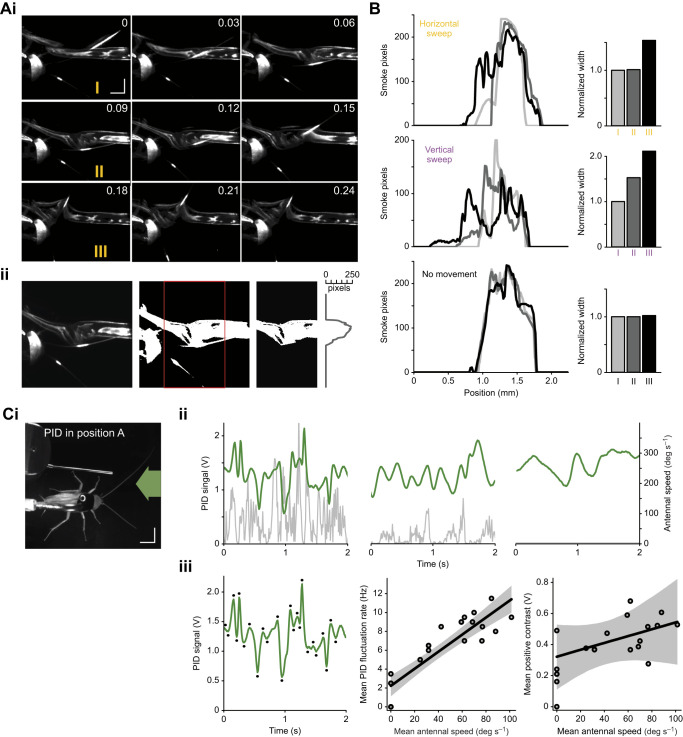
**Antennal movement affects the distribution and dynamics of air-borne plumes.** (A) Visualised air flow with TiO_2_ smoke. (i) Selected frames from a recorded sequence containing a horizontal antennal sweep through the plume. Time stamps are in seconds; scale bars: 10 mm. The inward movement is seen between 0.03 and 0.09 s, the outward movement between 0.12 and 0.21 s. (ii) Illustration of the analysis process to estimate smoke distribution. From left to right: raw frame; thresholded frame with the region of interest in front of the head marked by the red rectangle; removed antennae and limbs and the final distribution of smoke pixels within. (B) Smoke distributions for the three time stamps labelled I–III (*t*=0 s, *t*=0.09 s, *t*=0.18 s) in Ai, with their corresponding normalised widths, extracted from the frames of interest as illustrated in Aii. The width of frame I in each case was used to normalise the others for comparison. Shades of grey represent time stamps (I–III). Top: frames during the horizontal sweep shown in A. Frame I is before the sweep, frame II is at the end of the inward motion, and frame III is at the end of the outward sweep. Middle: a fast vertical sweep shown in Fig. S4B. Here, frame I is before the sweep, frame II is at the end of a full sweep cycle and frame III is 0.09 s afterwards. Bottom: a control sequence with a static antenna in the smoke plume (Fig. S4C). (C) Simultaneous antennal movement tracking and PID recording as a proxy for odour fluctuations. (i) Recording setup showing the PID in position A, the animal and the odour stimulus. Scale bars: 10 mm. (ii) Example recordings of the PID signal in green and simultaneous angular antennal speed in grey, for three different antennal movement regimes (from left to right): fast, slow and a stationary control with a fixed antenna. (iii) Left: an example PID trace with automatic peak detection to estimate rate and amplitude of odour fluctuations. Middle: correlation between the mean PID voltage peak rate and mean antennal speed (each point is the mean of a 2 s time window). Right: correlation between the mean positive voltage contrasts (signal increase from a negative peak to the next positive peak) and the mean antennal speed (same 2 s windows). In both, solid lines show linear regression curves with their shaded credible intervals (*n*=22).

Finally, we addressed the impact of antennal movement on the odour environment. We started with a simple quantification of the plume structure in different movement regimes. This was done by simulating an odorant-carrying air flow at low speed with light smoke. A planar infrared laser was used to visualise a horizontal section of the stream at the level of the animal, thus highlighting the effect of antennal movements on the air flow (see Materials and Methods, and example snapshots in Fig. 6A and Fig. S4B,C). We found that both slow horizontal and fast vertical sweeps affected the smoke distribution (top and middle panels in Fig. 6B show the distribution of smoke, before, during and after a horizontal and vertical sweep, respectively). The antennae, by successively passing back and forth through the stream, leave behind gaps when capturing smoke particles in their wake. The resulting distribution of the smoke after even a single sweep cycle is altered (Fig. 6A,B), an effect that lasts beyond the end of the sweep (Fig. S4B, see also Movie 2 showing the recording in Fig. 6A). To further evaluate the movement impact on odour dynamics, a PID was placed in the plume (Fig. 6Ci), close to the proximal third of the antenna, where the density of olfactory receptors is high (Paoli et al., 2020). Continuous recordings of PID voltage traces, a proxy for odour concentration (Fig. 6Cii, green traces), with the corresponding antennal kinematics (Fig. 6Cii, grey traces) revealed that odour fluctuations increase with increasing antennal movements. Peak-detection analysis of the PID traces (marked by black dots in Fig. 6Ciii) demonstrated that concentration fluctuations increased in rate (mean instantaneous rate of peaks) and amplitude with increasing antennal speed [Fig. 6Ciii; summarised statistics are given in Table S1B, including measurements from a more anterior PID position ahead of the antenna (2 cm in front of position A), showing a consistent, albeit weaker, speed-dependent trend]. Taken together, these findings highlight that antennal movements impact the rate of odour encounter, and that they do so in two different ways: by changing their position and sweeping pattern within the odour environment, but also by altering the structure of the odour environment itself.

## DISCUSSION

In this study, we investigated the use of antennal movements in olfactory sensing. We show that cockroaches locate and track static and moving odours by shifting the antennae towards the plume centre where local high-frequency movement bouts are carried out. Interestingly, however, long-range scanning sweeps continue throughout, such that the overall sensory range covered is not significantly impacted. Consistent with previous findings, during intermittent pausing bouts (potentially akin to the slow walking movements in this study), cockroaches displayed wide uncorrelated vertical and horizontal movement, presumably favouring the discovery of environmental cues (Okada and Toh, 2004). With odour presentation, cockroaches displayed changes in behaviour in an odour- or valence-dependent manner: enhanced antennal movement and decreased walking speed for attractive odour stimulation (sex-pheromone in Nishiyama et al., 2007, and colony odour here), which are inverted for an aversive odour stimulus (Nishiyama et al., 2007). Our current study did not include highly aversive odours, but the weaker antennal responses (Figs S2B, S3C) and slight increase in walking speed (Figs S1C, S3A) in response to our low valence odour (linalool) nevertheless indicate an overall consistent antennal sampling behaviour (Figs 3, 4Biii,Ciii, 5). Upon odour detection, a general increase in fast movement components was observed, with rapid vertical strokes exhibited most prominently during the stimulus presentation, and increased horizontal sweeps that also continued after stimulus termination. A similar increase in oscillation frequency has recently been reported in bumblebees (Claverie et al., 2023), corresponding to frequencies of optimal odour capture rates (Claverie et al., 2022). Utilising both numerical simulations and particle image velocimetry (PIV) experiments with artificial antennae, Claverie et al. (2022) (see also Su et al., 2019, and Reidenbach et al., 2008) demonstrated that the air flow vortexes generated by antennal oscillations can enhance the transport and adsorption of odour molecules through the sensory pores. In agreement with that, our *in vivo* visualisations of the flow and odour concentrations around the cockroach antennae provide empirical evidence that these thin appendages create significant flow fluctuations. Additionally, our results of increased movement frequency with odour detection are reminiscent of the antennal behaviour towards tactile stimuli (Okada and Toh, 2006), where an increase in contact frequency occurred with object detection, indicating an increased accuracy of orientation toward the object.

### Exploration–exploitation trade-off

Our first aim was to investigate how antennal movement participates in spatial sampling. It has recently been shown that honeybees orient their antennae towards appetitive odour sources and away from aversive ones (Cholé et al., 2022; Gascue et al., 2022). Moreover, the analysis demonstrated a general trend of positive correlation between the time the antennae spent focused on an odour target and its valence (Gascue et al., 2022). Similarly, we also observed that cockroaches shift the sweeps of at least one antenna towards the stream of appetitive odorants (exemplified by a decrease in distance to the odour stream, regardless of the stream's physical location). Although the behavioural differences between positions 2 and 3 and the strong correlation of the ipsilateral antenna with the odour movement (Fig. 4; Fig. S3, blue antenna) are in line with the spatial encoding of odours by a single antenna (Lockey and Willis, 2015; Nishino et al., 2018), we can only provide strong evidence for bilateral comparisons as a mechanism of encoding odour location (Fig. 3E–G, the left antenna reaches toward the right side upon odour encounter by the right antenna).

In terms of optimising the information animals can extract from their environment, there is an interesting interplay between sensing, or localising, a particular source of interest (‘exploitation’), and scanning the rest of the sensory space (‘exploration’). Our observations suggest that for antennal movement, this trade-off can be resolved with asymmetric sweeping, such that one antenna in particular spends more time in the odourised region, thus sampling the odorants more than the other. Regardless of the stimulus position, for both stationary and moving odours, the sweeping ranges and locations of the antennae are adjusted such that the overall scanning range (and therefore the possibility of detecting other stimuli elsewhere) and odour contact are maintained. It should be noted that there is no strict ‘division of labour’ between exploration and exploitation, as even the wide-sweeping contralateral antenna reaches towards the odour location in our asymmetrical experiments. However, the increase in sweeping range of that antenna (absent in the ipsilateral one) ensures coverage of the entire available space. This behavioural asymmetry, manifested differently depending on whether the stimulus is central or not, may offer a configuration for an efficient compromise between local sensing and larger-range scanning. We observed these sampling strategies in slow or intermittently walking tethered animals. It remains to be studied how this asymmetric antennation behaviour manifests during free-walking scenarios, where the different motion components [zig-zagging up-wind walking (Talley et al., 2022; Willis and Avondet, 2005) and antennation] layer and possibly compensate for each other for efficient evaluation of the odour environment. During flight, as air-flow fluctuations are increased and odour dynamics vastly differ from the boundary layer on the ground, the relative roles of these motion components may be entirely altered. In this scenario, flight trajectories play a major role in efficient odour sensing (Grünbaum and Willis, 2015; Liberzon et al., 2018; Talley et al., 2022) and antennal motion is suppressed and odorant–receptor interactions are predominantly entrained by wing beating (Chapman et al., 2018; Li et al., 2018; Loudon and Koehl, 2000).

### Odour intermittency and parallels to sniffing

Temporal dynamics of odour encounters depend on both the spatio-temporal structure of the odour environment and the way in which an organism moves, interacts and potentially reformats its olfactory surrounding (Crimaldi et al., 2021; Lei et al., 2022). Peripheral olfactory circuits allow the fast temporal encoding of intermittency and fluctuations in odour concentration (Egea-Weiss et al., 2018; Lemon and Getz, 1997; Szyszka et al., 2014) which have been shown to tune the neural activity in the antennal lobe (Huston et al., 2015; Lei et al., 2009; Martelli and Fiala, 2019) and were suggested to improve up-wind odour-source localisation (Lei et al., 2009; Willis and Baker, 1984). In addition to natural fluctuations, animals often display small oscillatory movements on top of their primary task-related trajectories (Chen et al., 2020; Liberzon et al., 2018; Willis and Avondet, 2005), which are intensified with novel stimuli detection (Huston et al., 2015; Loudon and Koehl, 2000; Schmitt and Ache, 1979). These behaviours enhance odour reception by imposing frequency-dependent fluctuations and creating sharp odour onsets at the olfactory organs, thus entraining odorant–receptor interactions (antennule ‘flicking’ in lobsters: Koehl et al., 2001; wing-flapping in flying insects: Li et al., 2018; Loudon and Koehl, 2000; Tripathy et al., 2010; and antennal movement: Claverie et al., 2022; Huston et al., 2015). In addition, olfactory processing may be modulated via specific neural circuitry projecting from motor networks to olfactory processing centres, further indicating that the movement itself could entrain odour encoding (Bradley et al., 2016; Chapman et al., 2017, 2018). We show here that cockroaches not only display comparable odour-induced modulations of antennal movement (Fig. 5) but also that these can impact the spatio-temporal structure of the odour environment (Fig. 6; Fig. S4). This could be of particular importance in low-turbulence and low-odour intermittency environments where cockroaches typically dwell. These elements can be paralleled with mammalian sniffing (Verhagen et al., 2007). During sniffing, the sharp input events, created through the rapid flow of odorants into the nasal cavity, have been shown to enhance odorant detection and discrimination (Kepecs et al., 2007; Oka et al., 2009). The sniffing frequency is controlled on a cycle to cycle basis according to task, the complexity of the olfactory environment and the animal's internal state (Verhagen et al., 2007), and increases upon odour encounter (Bhattacharjee et al., 2019; Reisert et al., 2020). At low to intermediate sniffing rates, intermittency in odour concentration allows receptor neurons to recover from adaptation during the intervals between successive inhalations Wachowiak (2011). With increases in sniffing frequency, seen during active exploration, signal attenuation also increases, resulting in enhanced contrasts for target odorants (temporally dynamic or spatially localized) against the broadly distributed background odours (Verhagen et al., 2007; Wachowiak, 2011). Additionally, and similar to motor-to-sensory neural pathways in insects (Chapman et al., 2018), there is evidence that the breathing rhythm itself could act as a coupling mechanism between neural networks (Karalis and Sirota, 2022). Parallels in the functional neural architecture of different olfactory systems (Strausfeld and Hildebrand, 1999; Touhara and Vosshall, 2009) further suggest that movement-driven temporal patterning in arthropods could similarly impact odour coding in a frequency-dependent manner.

## Supplementary Material

10.1242/jexbio.245337_sup1 Supplementary information
