# Supplementary material for: Active smelling in the American cockroach
Source: J Exp Biol. 2023 Nov 8;226(21):jeb245337. doi: 10.1242/jeb.245337 (PMC10651109; doi:10.1242/jeb.245337)
Supplement: Supplementary information [file jexbio-226-245337-s1.pdf]

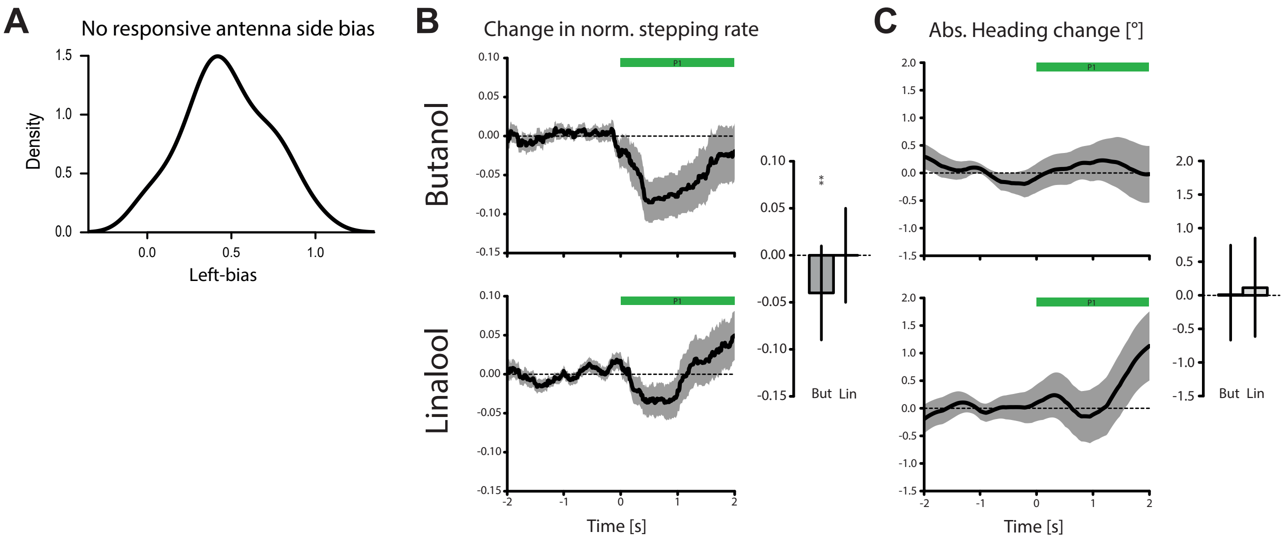

Fig. S1. Supplementary figure: Odour valences, response side bias and general odour-induced movement responses (A) Distribution of left-biases for the identity of the responsive antenna across trials for all odours, within animals: $Lbias_{animalID} = N_{Lresp}/(N_{Lresp} + N_{Rresp})$ (n=138 trials, N=26 animals). (B) Change in normalised stepping rate (n=38 trials, N=27 animals for Butanol; n=45 trials, N=24 animals for Linalool). (C) Change in absolute heading (n=46 trials, N=30 animals for Butanol; n=48 trials, N=28 animals for Linalool). Mean time-series ± s.e.m. and model means with credible intervals. In (B)-(C), asterisks indicate the certainty levels of the means to be different from zero (*: $\geq$ 90%, **: $\geq$ 95%, ***: $\geq$ 99%)

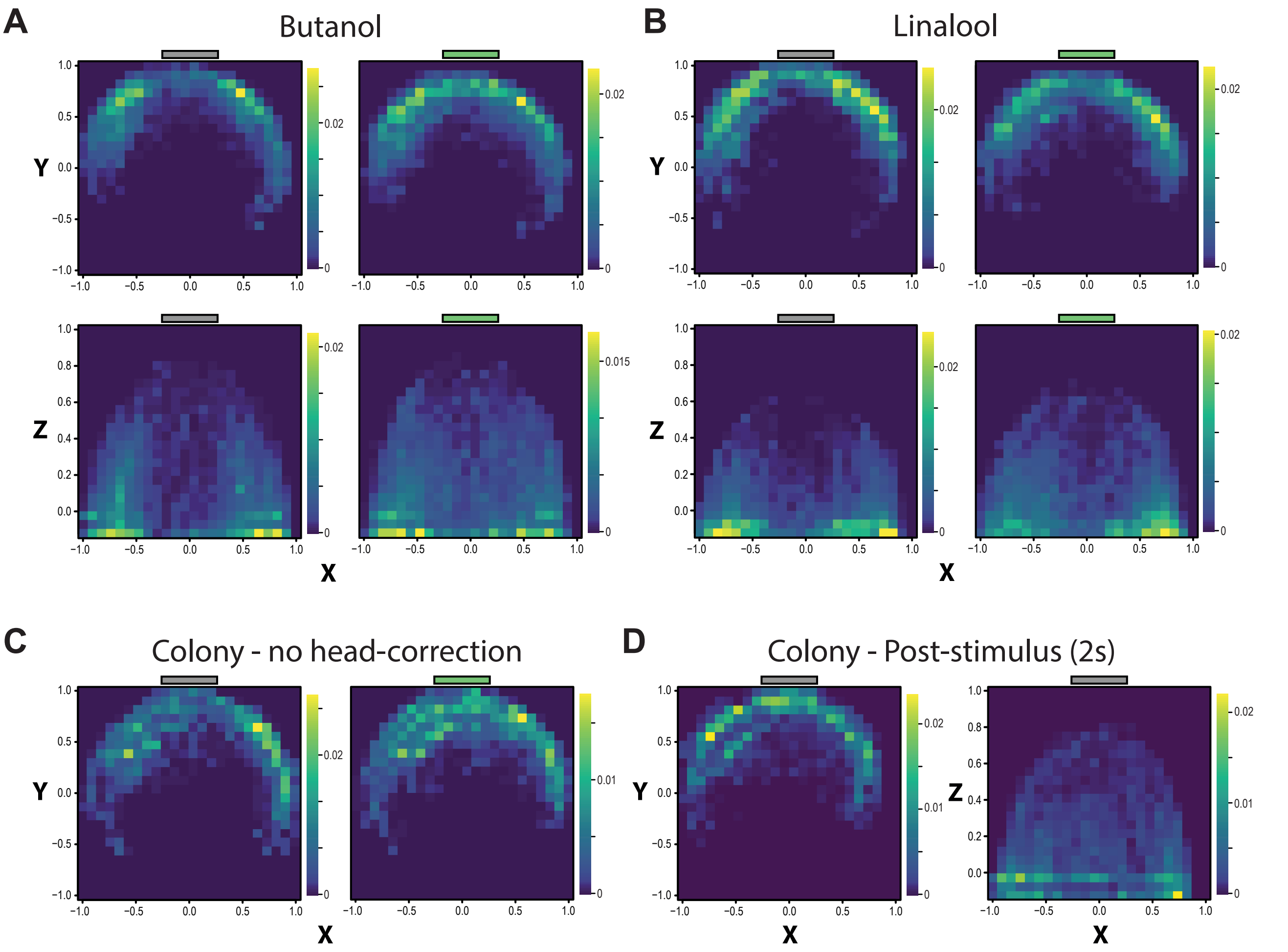

Fig. S**2.** General odour-induced antennal positions (A) Top row: Horizontal antennal tip density maps in the normalised coordinate system (zero = head position, ±1 = maximum coordinate across trials) before (left, grey bar) and during (right, green bar) a butanol stimulus (n=46 trials). Colours indicate relative proportions of observations per pixel. Bottom row: Same thing for the vertical antennal tip presence density in the normalised coordinate system (zero = head position, 1 = maximum coordinate across trials, 0 = ground). (B) Same as in (E) for a linalool stimulus (n=48 trials). (C) Same as in Fig.2Ci for the colony stimulus, but without head-centred antennal coordinates. (D) Pooled post-stimulus density maps for the colony odour, in the horizontal plane and the vertical plane. Same method as above and in Fig. 2C-D.

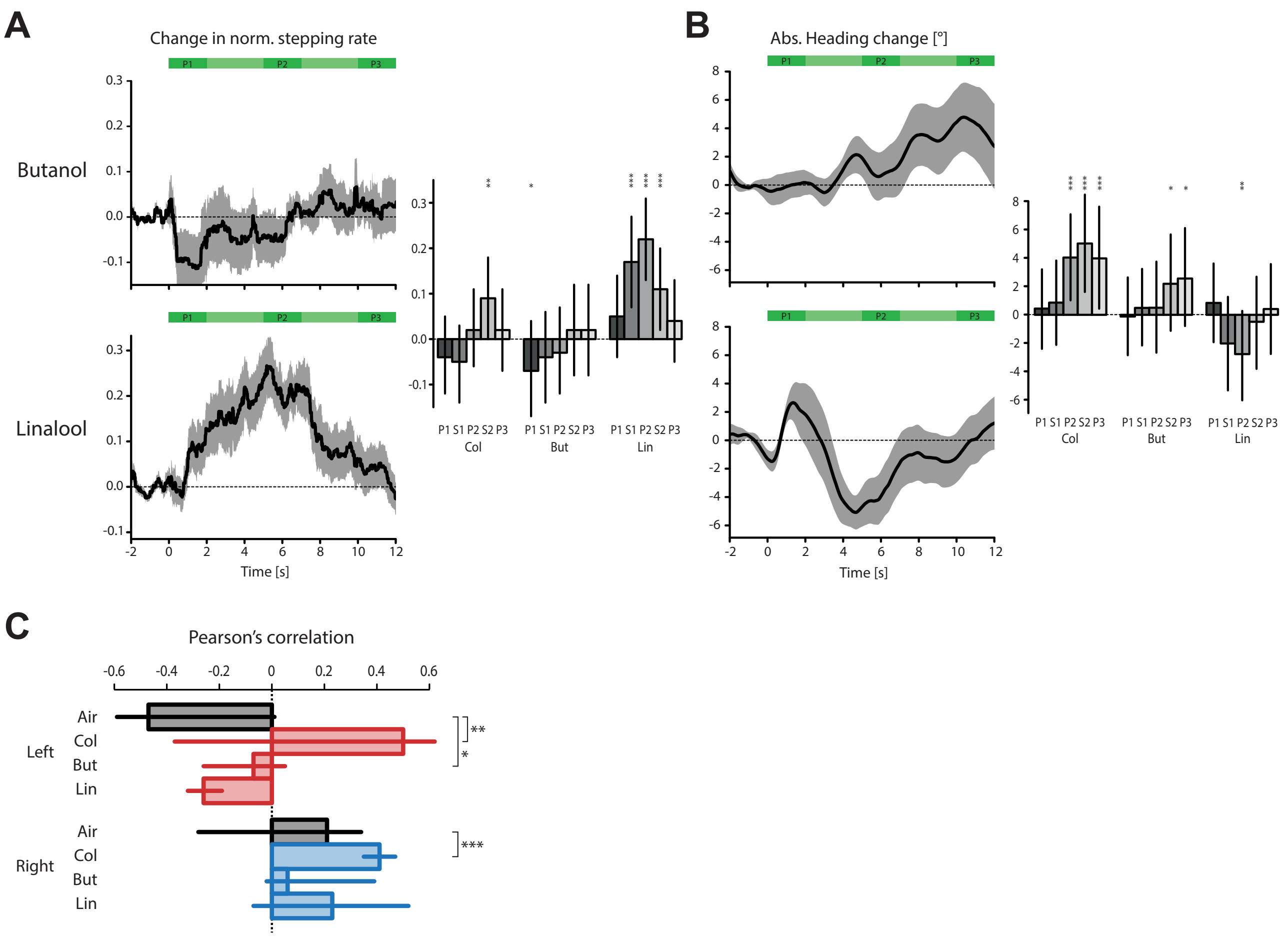

Fig. S3. Body and antennae movement responses during shifting odours. (A) Change in normalised stepping rate (n=13 trials for Colony; n=10 for Butanol; n=12 for Linalool). (B) Change in absolute heading (n=13 trials for each). Mean time-series ± s.e.m. and model means with credible intervals for each odour and each static position (P1-3). Asterisks indicate the certainty levels of the means to be different from zero (*: ≥ 90%, **: ≥ 95%, ***: ≥ 99%). (C) Pearson's correlation coefficient between the antennal position (the "centre of sweeps" corresponding to the average X coordinate at a given time point, calculated with a moving average) and the centre of stream for each antenna and each odour, as well as the air sham control (N=13 trials for each). Asterisks indicate the certainty levels of the antennal means to be different from their respective controls (*: ≥ 90%, **: ≥ 95%, ***: ≥ 99%).

Journal of Experimental Biology • Supplementary information

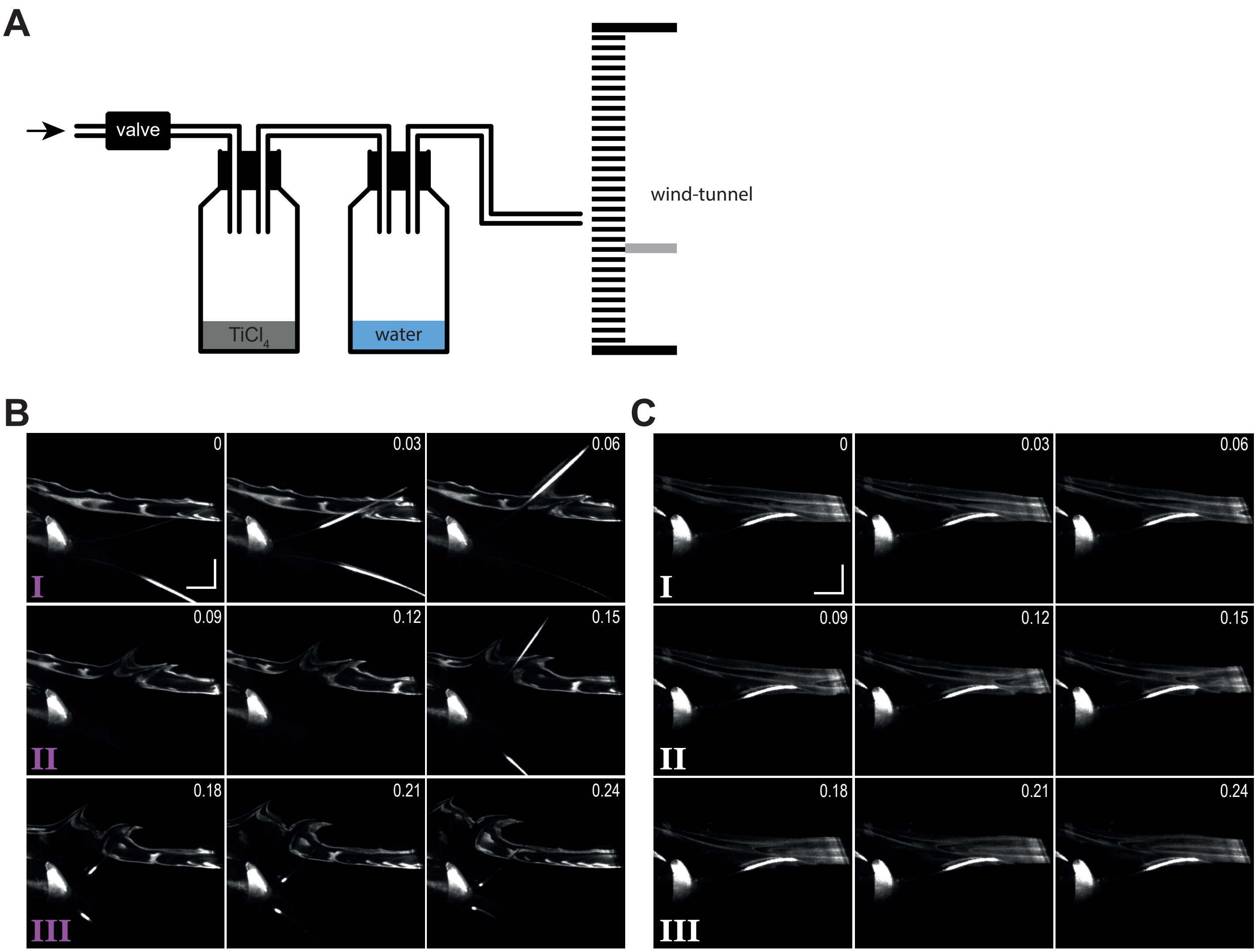

Fig. S**4. Odour plume visualisations** (A) Schematic of the smoke delivery set-up. (B) Visualised air flow with TiO_2 smoke. Selected frames from a recorded sequence containing a vertical antennal sweep through the plume. Time-stamps are in seconds, scale bars equal 10mm. The upward movement is seen at 0.03 and 0.06s, the downward movement is already completed at 0.09s. Frames labelled I-III are analysed in Fig. 6B.(C) Same thing with a dead animal for the 'no movement' control.

Table S1. (A) Model parameters. (B) Statistics for PID fluctuation metrics.

A

Response	Fixed effects	Random effects	Response distribution	Data pre-transformation
Fig. 2B: Change in stepping rate / Abs. heading	Window (during/after); Odour (Col/But/Lin)	Animal ID	Student (link = identity)	_
Fig. 2Cii				
Fig. 2Dii: Number of antenna observations	Location (above/below); Window (during/after)	_	Normal (link = identity)	_
Fig. 3C: Change in distance (responsive)	Odour (Col/But/Lin)	Animal ID	Student (link = identity)	_
Fig. 3C: Change in distance (non-resp.)	Odour (Col/But/Lin)	Animal ID	Student (link = identity)	_
Fig. 3C: Change in range (responsive)	Odour (Col/But/Lin)	Animal ID	Student (link = identity)	_
Fig. 3C: Change in range (non-resp.)	Odour (Col/But/Lin)	Animal ID	Student (link = identity)	_
Fig. 3D: Change in overall range	Odour (Col/But/Lin)	Animal ID	Student (link = identity)	_
Fig. 3G: Change in distance	Odour (Col/But/Lin); Antenna (L/R)	Animal ID	Student (link = identity)	_
Fig. 3G: Change in range	Odour (Col/But/Lin); Antenna (L/R)	Animal ID	Student (link = identity)	_
Fig. 3H: Change in overall range	Odour (Col/But/Lin)	Animal ID	Student (link = identity)	_
Fig. 4: Pearson's correlation	Stimulus Type (Air/Odour)	Animal ID	Student (link = identity)	_
Fig. 4Bii: Corrected change in distance	Antenna (L/R); Position (1-3)	Animal ID	Student (link = identity)	_
Fig. 4Biii: Avg corrected change in distance	Odour (Col/But/Lin)	Animal ID	Student (link = identity)	_
Fig. 4Cii: Corrected change in range	Antenna (L/R); Position (1-3)	Animal ID	Student (link = identity)	_
Fig. 4Ciii: Corrected change in overall range	Position (1-3)	Animal ID	Student (link = identity)	_
Fig. 4ciii: Avg corrected change in overall range	Odour (Col/But/Lin)	Animal ID	Student (link = identity)	_
Fig. 5C: ΔP/P	Window (during/after); Frequency ranges ((0.5,1],(1,3],(3,5], (5,10])	Antenna (L/R) nested in Trial	Student (link = identity)	ln(x+1)
Fig. 6Ciii: PID fluctuation rate	Antennal speed	_	Normal (link = identity)	_
Fig. 6Ciii: PID positive contrast	Antennal speed	_	Normal (link = identity)	_

B

	Metric	Mean slope	P(>0)
PID in pos. A (Fig. 6Ci)	Mean fluctuation rate	0.0850554	>0.99
	Mean contrasts	0.0022004	0.8771
PID in pos. B (2cm anterior)	Mean fluctuation rate	0.0027992	0.8609
	Mean contrasts	0.0001765	0.7807

Journal of Experimental Biology • Supplementary information

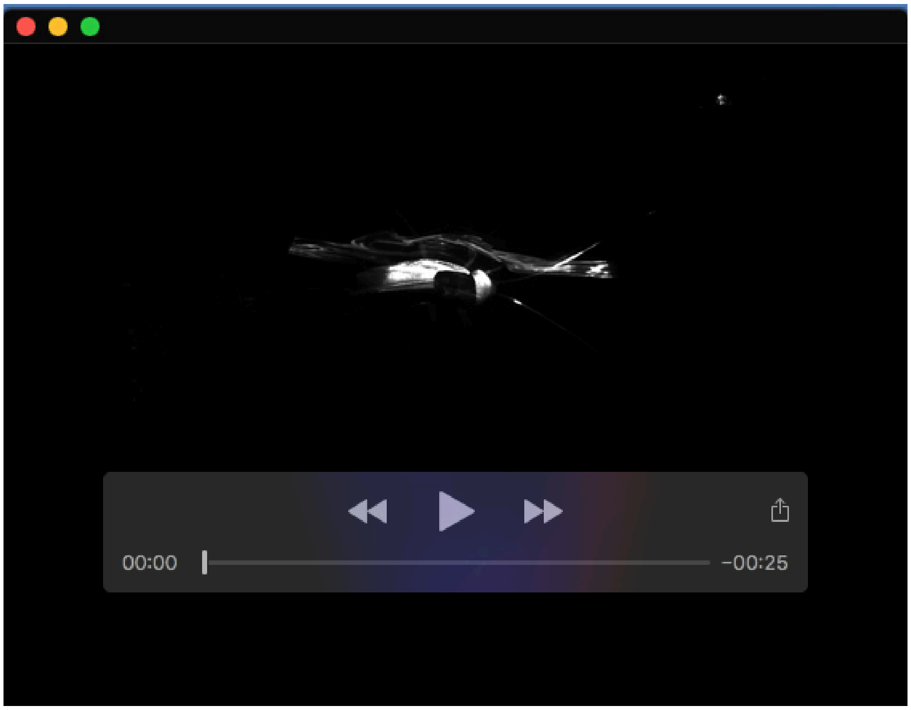

Movie 1. Example recording of the data shown in Fig. 1A. It shows the three synchronised recorded views (top, left, and right) of a tethered cockroach, starting shortly before odour onset until shortly after offset. The time of the odour stimulus is indicated by the infrared L.E.D. visible in the top right corner. The movie playback is set to half the real life speed.

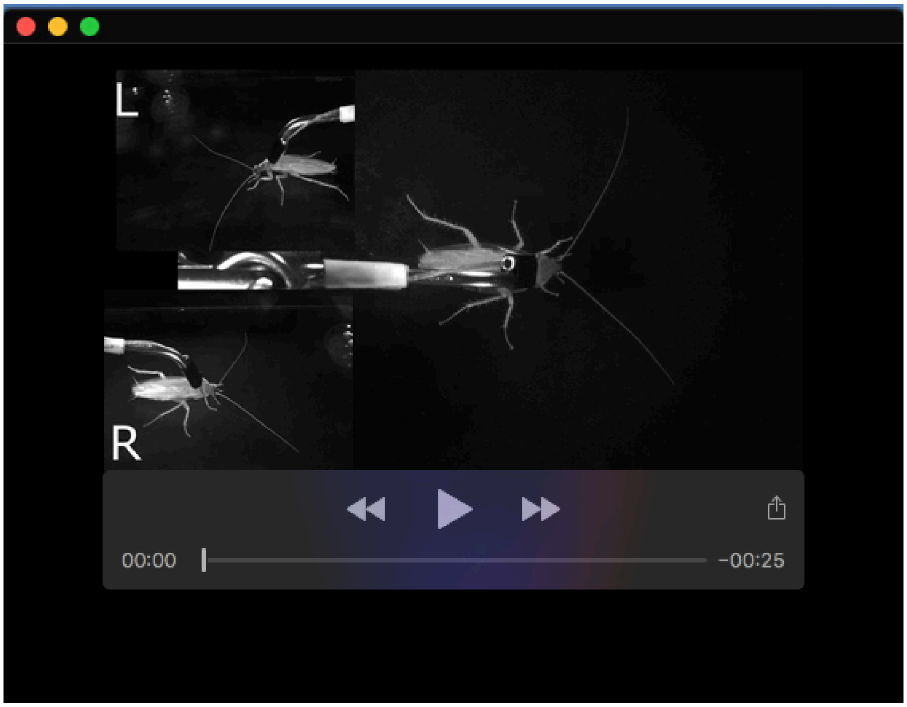

Movie 2. Video recording of the frame sequence shown in Fig. 6A. TiO2 smoke is being delivered from the right side and a planar infrared laser highlights a horizontal section of the scene. The cockroach is tethered on the left, facing the smoke in the same position as in movie 1. This recording shows a full horizontal sweep of the antenna through the smoke stream. The movie playback is set to half the real life speed.
